# A Novel Tissue and Stem Cell Specific TERF1 Splice Variant Is Downregulated in Tumour Cells

**DOI:** 10.3390/ijms21010085

**Published:** 2019-12-20

**Authors:** Yousef Ashraf Tawfik Morcos, Gregoire Najjar, Sabine Meessen, Britta Witt, Anca Azoitei, Mukesh Kumar, Gamal Wakileh, Klaus Schwarz, Hubert Schrezenmeier, Friedemann Zengerling, Christian Bolenz, Cagatay Günes

**Affiliations:** 1 Department of Urology, Ulm University Hospital, 89081 Ulm, Germany; yousefashraf93@gmail.com (Y.A.T.M.); gregoire.najjar@uni-ulm.de (G.N.); Sabine.Meessen@uniklinik-ulm.de (S.M.); Anca.Azoitei@uni-ulm.de (A.A.); mukesh.kumar@web.de (M.K.); Gamal.Wakileh@uniklinik-ulm.de (G.W.); Friedemann.Zengerling@uniklinik-ulm.de (F.Z.); Christian.Bolenz@uniklinik-ulm.de (C.B.); 2 Smartstep Consulting GmbH, 22081 Hamburg, Germany; britta.witt@smartstep-consulting.de; 3 Institute for Transfusion Medicine, University of Ulm, Ulm, Germany and Institute for Clinical Transfusion Medicine and Immunogenetics Ulm, German Red Cross Blood Service Baden-Württemberg – Hessen, 89081 Ulm, Germany; Klaus.Schwarz@uni-ulm.de (K.S.); h.schrezenmeier@blutspende.de (H.S.)

**Keywords:** telomere, TERF1, stem cells, spermatogoina, cancer

## Abstract

In this study, we describe the identification of a novel splice variant of TERF1/PIN2, one of the main components of the telomeric shelterin complex. This new splice variant is identical to TERF1, apart from a 30 amino acid internal insertion near to the C-terminus of TERF1. Based on genome comparison analyses and RNA expression data, we show that this splice variant is conserved among hominidae but absent from all other species. RNA expression and histological analyses show specific expression in human spermatogonial and hematopoietic stem cells (HSCs), while all other analyzed tissues lack the expression of this TERF1-isoform, hence the name TERF1-tsi (TERF1-tissue-specific-isoform). In addition, we could not detect any expression in primary human cells and established cancer cell lines. Immunohistochemistry results involving two new rabbit polyclonal antibodies, generated against TERF1-tsi specific peptides, indicate nuclear localization of TERF1-tsi in a subset of spermatogonial stem cells. In line with this observation, immunofluorescence analyzes in various cell lines consistently revealed that ectopic TERF1-tsi localizes to the cell nucleus, mainly but not exclusively at telomeres. In a first attempt to evaluate the impact of TERF1-tsi in the testis, we have tested its expression in normal testis samples versus matched tumor samples from the same patients. Both RT-PCR and IHC show a specific downregulation of TERF1-tsi in tumor samples while the expression of TERF1 and PIN2 remains unchanged.

## 1. Introduction

Telomeres are protective caps at the ends of human chromosomes that prevent loss of genetic material due to the end-replication problem and from end-to-end fusion of linear DNA ends discriminating them from double-strand breaks (DSB) [1,2,3]. Vertebrate telomeres are composed of the telomeric DNA, tandem repeats of the TTAGGG sequence (^~^3000 repeats at birth), the shelterin factors, a specific set of telomere-binding proteins, and TERRA (telomeric repeat–containing RNA), a non-coding RNA transcript [4,5,6]. The shelterin components include TERF1 (telomeric repeat-binding factor 1), TERF2 (telomeric repeat-binding factor 2), TIN2 (TERF1 interaction factor 2), RAP1 (repressor activator protein 1), ACD (ACD shelterin complex subunit and telomerase recruitment factor), and POT1 (protection of telomere 1) [6]. The shelterin proteins are associated with telomeric DNA to keep the telomere integrity and functionality, and to protect telomeric-DNA from DNA-degradation and from activating the DNA-damage signaling [7,8]. Extensive telomere shortening or the impaired function of shelterin components results in dysfunctional telomeres, leading to chromosomal instability [9,10,11,12,13,14], the context of tumourigenesis: in cells with intact checkpoints (i.e., functional p53 and Rb responses), telomere dysfunction induces cellular senescence or apoptosis providing a tumor suppressor mechanism against genome instability caused by too short or deprotected telomeres [15,16,17,18,19,20]. On the contrary, in the absence of check-point control mechanisms, dysfunctional telomeres can promote tumorigenesis by initiating breakage–fusion–bridge (BFB)-cycles [12,21,22,23,24,25,26].

Among the shelterin proteins, TERF1 and TERF2 directly bind the double-stranded telomeric DNA and collectively form a platform for the assembly of a higher-order telomeric structure for chromosome end-protection. Despite a high similarity of their protein sequences in the dimerization regions, TERF1 and TERF2 do not form heterodimers and have specific, different functions at the telomeres. Data indicate that TERF1 has a specific role in S-phase, important for replication fork movement, as loss of TERF1 results in a dramatic increase of fragile telomeres in S-phase [27]. On the other hand, ectopic expression of TERF1 results in telomere shortening, whereas depletion of TERF1 results in telomere elongation, indicating a central function of TERF1 in telomere length control [14]. Interestingly, a splice variant of TERF1, PIN2, was identified as an NIMA interacting protein [28] and was later found to be related to TERF1 [29]. PIN2 is identical to TERF1 except for a 20 amino acid internal deletion, produced by alternative splicing of TERF1/PIN2 gene. Although both, PIN2 and TERF1 are ubiquitously expressed among human cell lines and tissues tested so far, interestingly, PIN2 is more abundant than TERF1 in established cell lines [29]. It is also conserved among vertebrates, including mice. To date, no clear functional difference between PIN2 and TERF1 was found, although it was reported that PIN2 is abnormally concentrated only at a few telomeres in telomerase-negative cells [30,31,32]. Therefore, both splice variants are collectively described as TERF1/PIN2.

Here we describe the identification of a novel TERF1/PIN2 splice variant. Based on genome comparison analyses and RNA expression data, we show that this new splice variant is conserved in hominidae. RNA expression and histological analyses show that the expression is restricted to spermatogonial and hematopoietic stem cells (HSCs) in humans. Due to its tissue-specific expression, this isoform is referred to as TERF1-tsi, a 469 amino acid protein, characterized by an additional 30 amino acid internal insertion close to the C-terminus of TERF1 protein. Of note, TERF1-tsi expression was absent in all so far analyzed cell lines. Immunohistochemistry results with two new rabbit polyclonal antibodies, generated against TERF1-tsi specific peptides, indicate nuclear localization of TERF1-tsi in a subset of spermatogonial stem cells. Importantly, TERF1-tsi is expressed in normal testis samples but absent in matched tumor samples from the same patients indicating a potential role of TERF1-tsi in tumor suppression. Immunofluorescence analyzes in various cell lines consistently revealed that ectopic TERF1-tsi localizes to cell nucleus, mainly but not exclusively at telomeres.

## 2. Results

### 2.1. Expression Analyses of the Novel TERF1 Splice Variant

**Figure 1:**
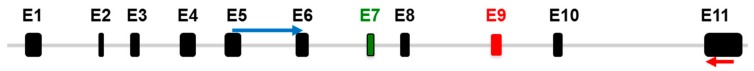
Schematic drawing of the intron-exon structure of *TERF1* genomic locus. Not drawn to scale. E1–E11 represent the exons, including exon 9 (red) which is found in the new isoform, described in this manuscript. E9 and E7 (green) are absent in the PIN2 splice variant while TERF1 lacks E9 only. The colored arrows indicate the location of the primer (blue, forward primer and red, reverse primer) which were used to amplify the three *TERF1* splice variants in a single PCR reaction (hmsFor and hmsRev, respectively).

**Figure 2:**
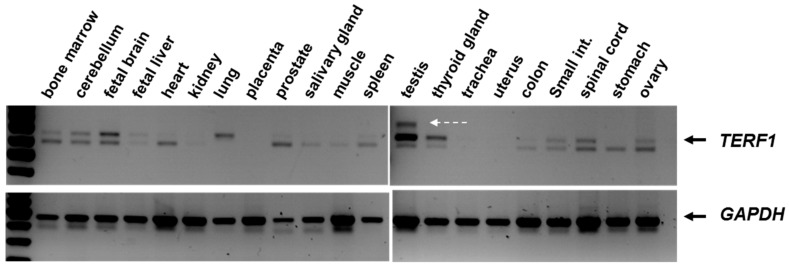
Semi-quantitative PCR analysis showing the *TERF1* splice variants (top) and *GAPDH* products (bottom). White arrow indicates the additional PCR product in the testis sample. PCR reaction was performed using the primer pair indicated in Figure 1. Of note, we observed variations in TERF1 and PIN2 splice variants among the tissues (e.g., absence of TERF1 in the stomach or absence of PIN2 in the lung tissue). Please also note that *TERF1/PIN2* mRNAs are not visible in the presented figure due to low signal intensity, although both splice variants are expressed in these tissues.

**Figure 3:**
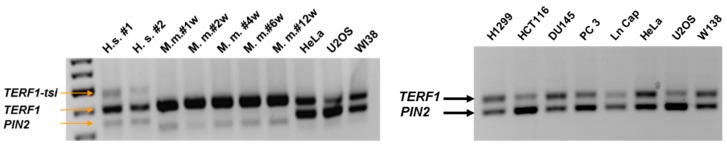
Semi-quantitative PCR showing expression of the *TERF1* splice variants in human and mouse testes and human cell lines. PCR reaction was performed using the primer pair indicated in Figure 1. Of note, we observed higher *PIN2* mRNA levels in the human cell lines in comparison to *TERF1*, while *TERF1-tsi* was not detectable in these cell lines. Also, note that the mouse *Pin2* is shorter compared to human *PIN2*. (Left) Human testis samples (H.s. #1 and H.s. #2), testis samples from mice (1, 2, 4, 6, and 12 weeks post-partum) and human cell lines are indicated. (Right) An independent RT-PCR reaction was performed using total RNA from indicated human cancer cell lines and the human primary cell strain WI38.

**Figure 4:**
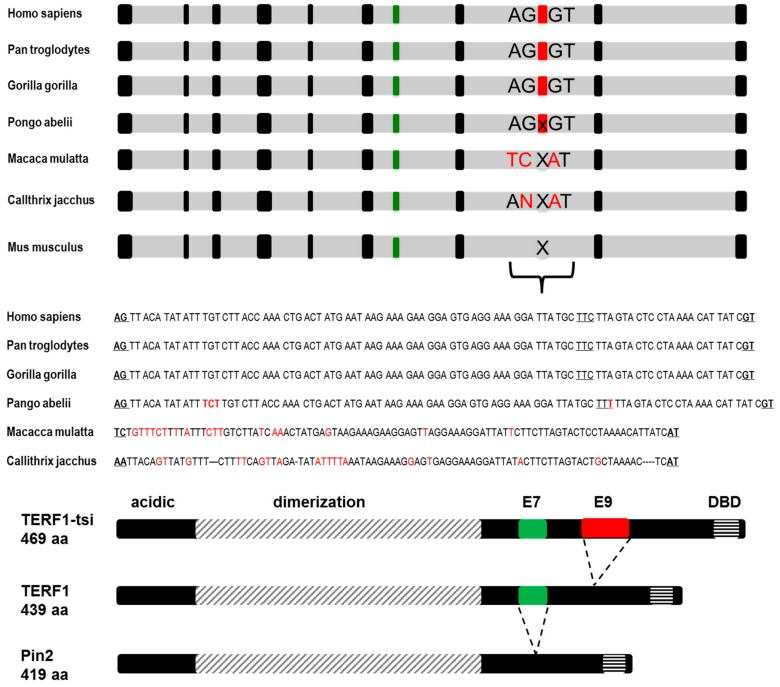
*TERF1-tsi* encompasses an evolutionarily conserved novel exon, exon 9. (Top) Schematic drawing of the intron-exon structure of *TERF1* genomic locus. Not drawn to scale. Exons 7 (green) and 9 (red) are color-indicated by the red rectangle. The lower-case x in the red rectangle (*P. abelii*) indicates high sequence similarity to human exon 9 whereas the upper-case X indicates strong sequence variation in this region. (Middle) Exon 9 and conserved AG-GT intron-exon junction target sequences of human, chimpanzee, gorilla, orang-utan are indicated in bold and underlined, along with the corresponding genomic regions of macaca and callithrix. Sequences varying from the conserved exon sequences are shown in red color. Of note, P.a. (orang utan) exon 9 has an additional triplet and thus an additional amino acid while keeping the reading frame. Moreover, there is a difference at the third Wobble position (C in human, chimpanzee, and gorilla versus T in orang utan) in the underlined triplet (red color) without a consequence of amino acid change (shown in red). The triplets encoding the amino acids are indicated. Please note that the first two and the last nucleotides of exon 9 are aligned to be in frame with TERF1 amino acid sequence. (Bottom) Drawing indicating the conserved domains of *TERF1* splice variants. The N-terminal acidic domain, the dimerization domain, and the DNA-binding domain (DBD) are shown. In addition, exon 7 (E7), which is missing in PIN2, and exon 9 (E9), which is only present in TERF1-tsi, are indicated by a green or a red rectangle, respectively.

**Figure 5:**
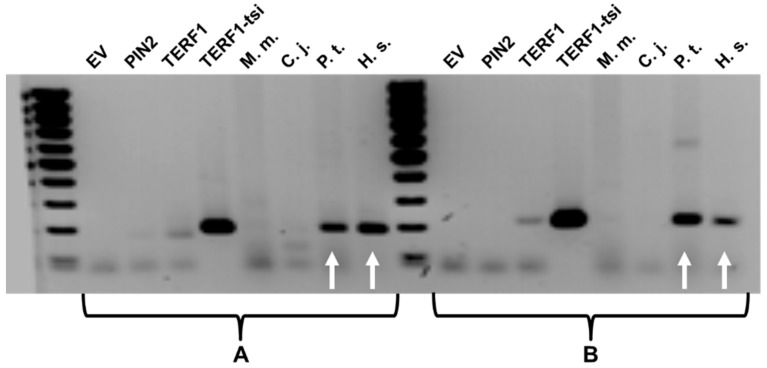
TERF1-tsi expression is observed in human and chimpanzee testis samples. Results from the semi-quantitative PCR showing the *TERF1-tsi* expression in testis samples from *M. musculus* (M.m.), *C. jacchus* (C.j.), *P. troglodyetes* (P.t.) and *H. sapiens* (H.s.). Please note that testis samples were not available from *G. gorilla* and *P. abelii*. PCR reaction was performed using two different *TERF1-tsi* specific primer pairs, primer pair A (left) and primer pair B (right), respectively. Plasmid DNA with cloned *PIN2*, *TERF1*, or *TERF-tsi* cDNA as well as empty vector (EV), were used to control the specificity of the primer. Due to high plasmid DNA concentrations used for the positive control PCR reactions, weak, non-specific PCR products were also visible with the TERF1 cDNA. White arrows indicate *TERF1-tsi* expression in human (H.s.) and chimpanzee (P.t) testis samples.

The sequencing result of the human genome indicated a potential splice variant encompassing a 90 base pair exon within the TERF1 genomic sequence (NC_000008.11). The automated computational analysis predicted an additional 30 amino acid sequence within the TERF1 protein sequence. We designed a primer pair to detect potential TERF1 splice variants, including the exon 7 (absent in PIN2 and present in TERF1) and the hypothetical exon 9 in one single PCR reaction in a variety of human tissue samples (Figure 1, Figure 2, Figure 3, Figure 4 and Figure 5). The forward primer spans over exons 5 and 6 while the reverse prime lies within exon 11, enabling the amplification TERF1 splice variants in the same PCR reaction, including the potential new exon 9 (Figure 1).

We used a set of commercially available RNAs extracted from 21 human tissue samples to detect *TERF1* splice variants, along with the *GAPDH* loading control in a semi-quantitative RT-PCR reaction. As expected, we detected *TERF1/PIN2* splice variants in all tissues analyzed here, although to varying levels. Interestingly, we observed an additional PCR product in the human testis sample, which was larger than both *TERF1* and *PIN2* (Figure 2, indicated with white arrow).

To exclude potential PCR artefacts, next, we performed the same RT-PCR reaction using cytoplasmic RNA purified from two independent human testis samples along total RNA prepared from cultured human cells and with mouse testes RNA prepared from 5 different mice (Figure 3). Again, we observed three different PCR products with human testis RNA samples, corresponding to *TERF1/PIN2* and an additional product, potentially corresponding to a novel splice variant. Of note, although expected *TERF1/PIN2* products could be detected in mouse testis samples and the human cell lines, no additional band was observed in these samples (Figure 3). The cloning and subsequent sequencing of this additional PCR product revealed a potential 90 bp exon, flanked by the conserved AG-GT intron-exon junction target sequences, in line with the suggested sequence of the hypothetical exon 9. We thus refer to this novel *TERF1* isoform as *TERF1-tsi* (*TERF1-tissue-specific-isoform*).

Prompted by these observations, we compared genomic sequences of the exon 9 and its flanking regions, especially focusing on the conserved AG-GT intron-exon junction target sequences among some primates (*H. sapiens, P. troglodytes, G. gorilla, P. abelii,* and *M. mulatta*) and mouse (*M. musculus*) (Figure 4). Interestingly, while both the intron-exon boundary sequences conserved among homidae, *P. abelii* (orang utan) has a CTT insertion close to the 5’-end of the exon (emphasized by large capital letters in Figure 4), causing a frame-shift in the hypothetical *TERF1* splice variant in this animal. Both the exon-intron boundary sequences and the hypothetical exon sequence are not conserved in *M. mulatta* or *C. jacchus* (Figure 4). Mouse *Terf1* genomic DNA lacks any sequence similarity for a potential *TERF1-tsi* splice variant.

To verify the results from genome comparison analysis for the potential expression of the new *TERF1* splice variant, a semi-quantitative RT-PCR was performed using RNA from available monkey testes (*P. troglodytes, M. mulatte*, and *C. jacchus*), in addition to human testes. For this purpose PCR conditions with two different exon-nine specific primer pairs were established and the specificity of the primer pairs was verified using plasmid vectors, including the cloned cDNA for the *TERF1* variants. Our results show that *TERF1-tsi* is expressed in human and chimpanzee testis samples but not in those of *M. mulatta* or *C. jacchus* (Figure 5).

### 2.2. Cell Type-Specific Expression of TERF1-tsi

**Figure 6:**
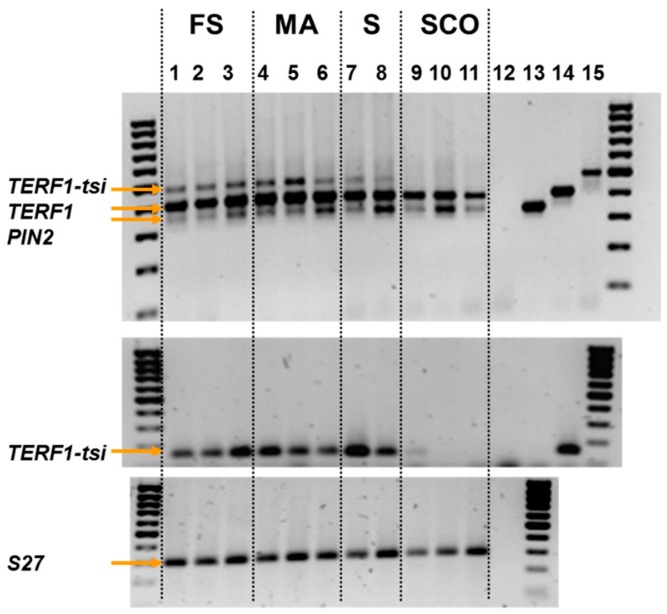
TERF1-tsi expression in pathological human testis samples. Semi-quantitative PCR. RNA was isolated from the indicated samples (FS: full spermatogenesis; MA: meiotic arrest; S: spermatogonia only; SCO: Sertoli cells only) and RT-PCR was performed. Plasmid DNA with cloned *PIN2*, *TERF1,* or *TERF-tsi* cDNA, as well as an empty vector, were used to control the specificity of the primer. Numbers 12–15 indicate vector controls (12: empty vector; 13: PIN2-vector; 14: TERF1 vector; and 15: TERF1-tsi vector). PCR reaction was performed using a pan-TERF1 primer (top) or a *TERF1-tsi* specific primer. Please note that the PIN2 and TERF1 vector controls were left out here. Numbers 12–14 indicate controls (12: H_2_O; 13: empty vector; 14: TERF1-tsi vector) (middle). Expression of the ribosomal gene S27 was used as a control (bottom). Please note that all vector controls were left out here. Number 12 indicates H_2_O control.

The above data clearly indicated testis-specific expression of *TERF1-tsi*, while TERF1/PIN2 are ubiquitously expressed. In an attempt to elucidate the cell type-specific expression of *TERF1-tsi* in detail, we examined its expression in a subset of human testis samples with differentiation defects and in testis samples with Sertoli cells only (see legend to Figure 6). Semi-quantitative RT-PCR analyses, both with pan-*TERF1* primer combination or specific for *TERF1-tsi*, indicated a lack of TERF1-tsi in Sertoli cells (Figure 6).

**Figure 7:**
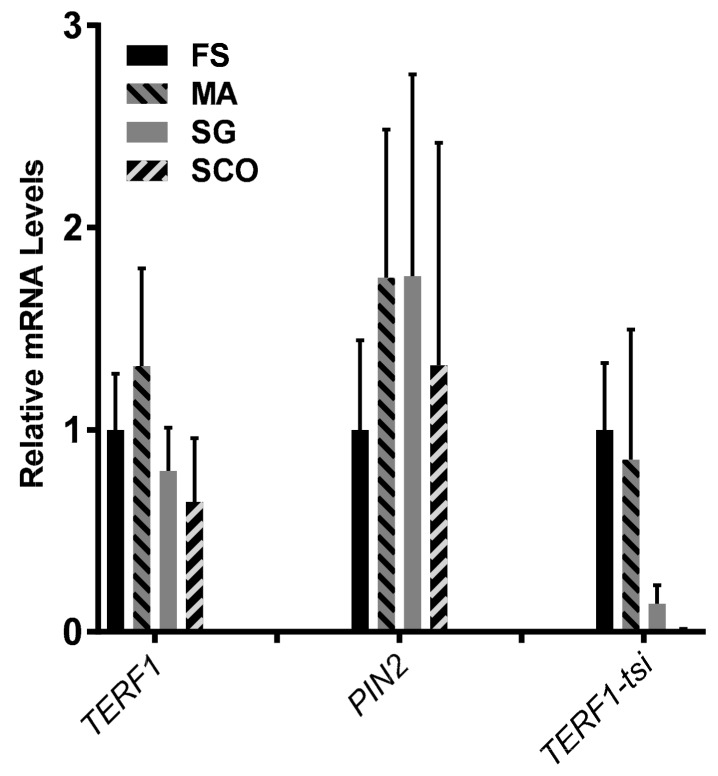
TERF1-tsi expression in pathological human testis samples. Real-time quantitative RT-PCR. RT-qPCR with isoform-specific primer pairs indicate the absence of TERF1-tsi in Sertoli cells. The same samples as in Figure 6 were analyzed in this experiment. Please note the absence of TERF1-tsi in the SCO samples. Also interesting is the relatively low expression of TERF1-tsi in SG samples. Real-time RT-PCR were mean values from two independent experiments with three technical replicates each. Error bars show mean of standard deviation (SD).

These results could be verified by quantitative real-time PCR analysis (Figure 7). Again, TERF1/PIN2 could be detected in all samples, while the expression of TERF1-tsi was absent in patient samples with Sertoli cells only.

**Figure 8:**
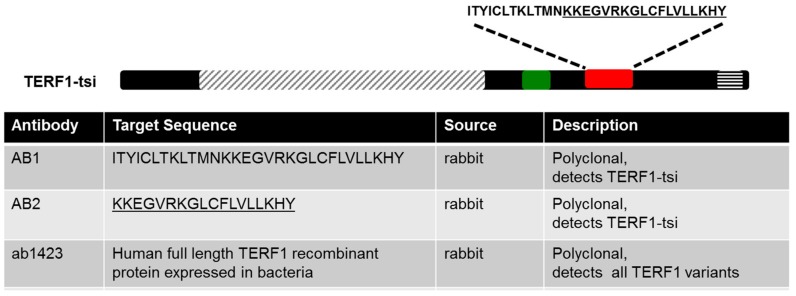
Anti-TERF1 antibodies used in this study. AB1 was raised against the full exon 9, and AB2 was raised against the underlined amino acids in exon 9 (red box). Ab1423 was purchased from Abcam. Green box indicates exon 7.

Although the RT-PCR results showed a lack of TERF1-tsi in Sertoli cells, we could not distinguish its expression among other cell types such as spermatogonia, spermatocytes, spermatids, and sperm cells. This question is addressed by immunohistochemistry (IHC) in the next set of experiments. Since commercially available anti-TERF1 antibodies would not discriminate between the TERF1 isoforms, we assigned two different companies to generate TERF1-specific polyclonal antibodies in rabbits by (Figure 8; see Section 4 for antibody generation).

**Figure 9:**
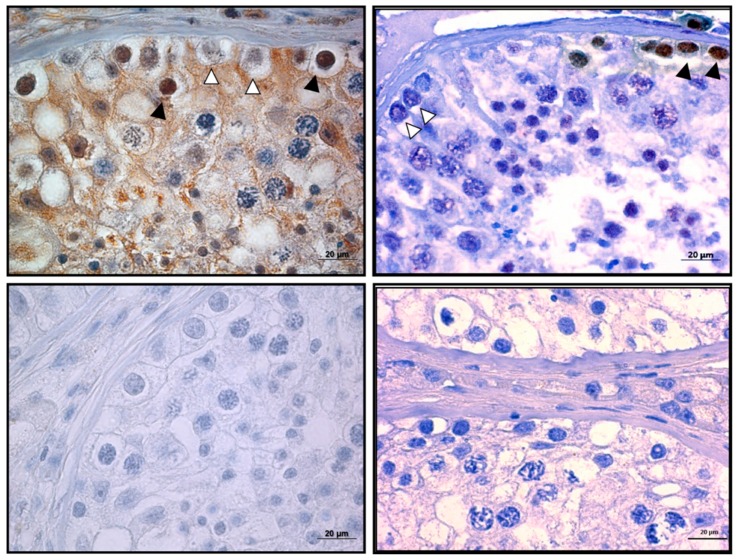
Immunohistochemical detection of TERF1-tsi in a human testis section with a specific TERF1-tsi antibody, AB2 (1:400). Representative image of testis showing a single seminiferous tubule, Leydig (interstitial) cells, and surrounding muscle cells is shown. AB2 showed strong positive staining (likely TERF1-tsi), specifically localized in spermatogonia, as shown in the top images (black arrowheads). Of note, some of the spermatogonia again did not show any staining (white arrowheads with black border). Importantly, no staining was observed with AB2 on mouse testis (Supplementary Figure S2), supporting the specificity of the AB2 antibody for human TERF1-tsi. This section was counterstained with hematoxylin. The images were captured at 200×. Scale bars (20 µm) are indicated.

**Figure 10:**
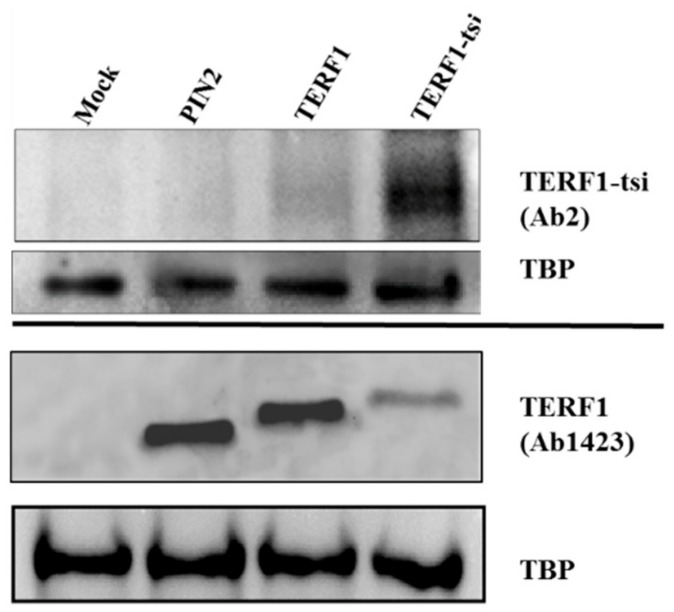
Western blot showing the specificity of AB2. HEK cells transfected with empty vector or with vectors expressing the indicated TERF1 variants and nuclear extracts were prepared. AB2 shows a strong TERF1-tsi signal in the respective nuclear extracts, though a weak background signal is also visible in TERF1 transfected cells. Independent immunoblot with the pan-TERF1 antibody ab1423 and the same nuclear extracts prove the expression of the transfected TERF1 variants. TBP was used as a loading control. Of note, despite the exact DNA amounts were used for transfection, TERF1-tsi protein levels were consistently low, potentially indicating post-transcriptional regulatory mechanisms to keep low TERF1-tsi levels. This was not due to promoter activity or DNA quality, as we observed this phenomenon consistently with two other vector systems (compare to Figure 5G and see discussion).

Antibody 1 (AB1), raised against a peptide mapping the full 30 amino acid of exon 9, and antibody 2 (AB2), raised against a peptide representing the C-terminal 18 amino acids of exon 9, were then used for IHC analyses. Both antibodies showed specific immunoreactivity in spermatogonia, though the AB1 antibody had higher background signals (Figure 9, left). Interestingly, the nuclear staining with both antibodies was restricted to a subtype of spermatogonia (Figure 9 and Figure S1). Importantly, IHC staining with pan-TERF1 antibody ab1423 clearly indicated the presence of TERF1/PIN2 in all cell types, including Leydig and surrounding muscle cells (Supplementary Figure S1). Though the antibodies were less sensitive for Western blot analyses, a specific signal was detected with AB2 in cells overexpressing the TERF1-tsi isoform (Figure 10). The expression of the ectopic proteins was also verified by the ab1423 antibody (Figure 10). Of note, compared to TERF1/PIN2 levels, a low TERF1-tsi protein amount was detected consistently. This observation was independent of transfection efficiency, plasmid purity/concentration or cell types, as it was observed with several independent vector systems, cell types, and transfection/infection methods, indicating post-translational regulation of TERF1-tsi in cultured cells (compare to Figure 18 and see discussion).

### 2.3. TERF1-tsi Expression Is Downregulated in Seminomas

**Figure 11:**
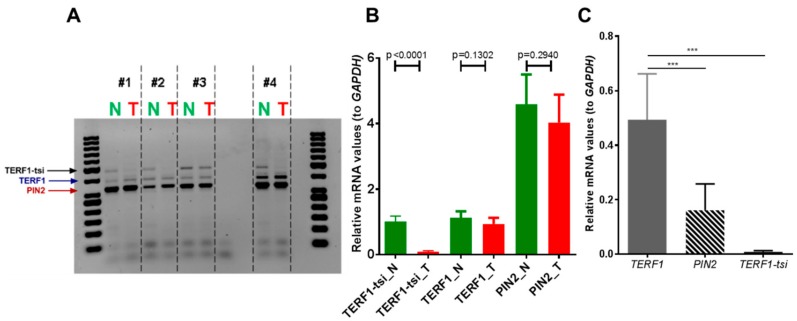
*TRF1-tsi* expression is absent in human seminomas and the TCam-2 seminoma cell line. (**A**) Semi-quantitative RT-PCR and (**B**) real-time quantitative RT-PCR showing specific downregulation of *TERF1-tsi* mRNA levels in testis tumors or (**C**) in TCam-2 cell line. In (A), pan-TERF1 primer pairs were used, whereas isoform-specific primer pairs were used in (B) and (C). Please note that RNA for normal (N) and tumor (T) samples #1, #2, and #3 were purchased commercially (see Section 4: Materials and Methods) while #4 was prepared from a patient at Ulm University Hospital. Of note, while tumors #1 and #2 were seminoma samples, #3 was defined as intratubular germ cell neoplasia. Matched N/T samples #4 were also embedded and analyzed by immunohistochemistry in the subsequent experiments (e.g., Figure 12). Real-time RT-PCR were mean values from two independent experiments with three technical replicates each (n = 4). Error bars show the mean of SD. *p*-Values were calculated by unpaired *t*-test with Welch’s correction. *** indicates *p* = 0,0005.

**Figure 12:**
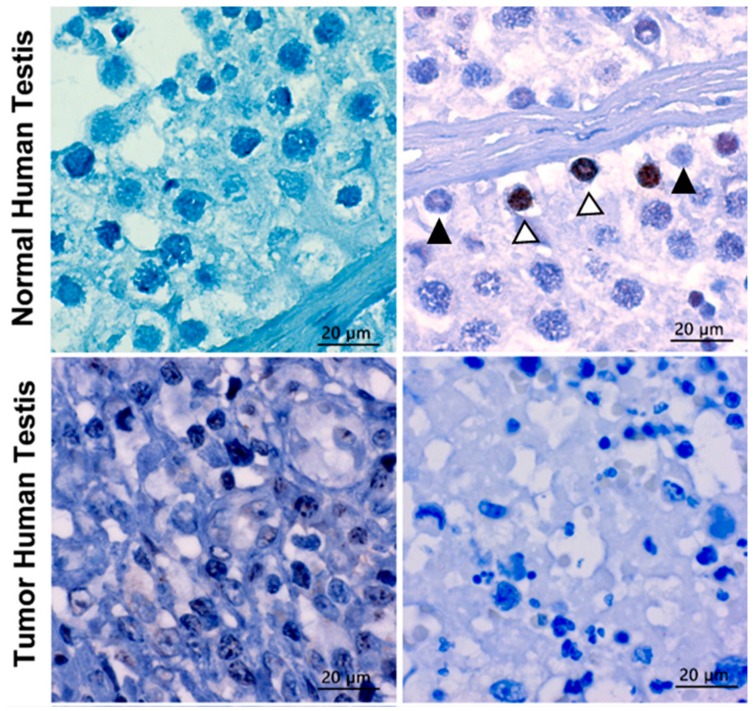
TERF1-tsi protein is downregulated in human seminoma sample. (Left) Top and bottom left figures demonstrate the IHC analysis of negative controls (only secondary antibody) of normal and tumor testis, respectively, showing no background staining. (Right) The upper figure depicts the IHC analysis of TERF1-tsi in normal human testis with AB2 showing positive staining (TERF1-tsi) in a subset of spermatogonia (white arrowheads with black border). Of note, some of the spermatogonia did not show any staining (black arrowhead). In contrast, the bottom right figure demonstrates the lack of TERF1-tsi in tumor testis tissue with AB2, confirming the previous mRNA expression results (3A, #4 normal and tumor samples). All sections were counterstained with hematoxylin comparing and captured at 630×. The bar indicates 20 µm.

Previous publications indicate the deregulated expression of TERF1 in cancer [33,34,35]. In order to evaluate the potential contribution of the TERF1-tsi to testis cancer, we analyzed its expression by RT-PCR and IHC. Both the semi-quantitative RT-PCR using the primer pair which detects all *TERF1* variants (Figure 11A) and the real-time RT-qPCR using the isoform-specific primers (Figure 11B) show a clear downregulation of *TERF1-tsi* in seminoma tumors in comparison to the matched normal tissue sample from the same individual while *TERF1* or *PIN2* mRNA levels are not significantly different. Interestingly, weak TERF1-tsi downregulation was observed in testicular cancer sample #3, which is histologically defined as intratubular germ cell neoplasia (ITGN). ITGN are regarded as a precursor to invasive germ cell tumors of the testis and thus can contain undifferentiated/non-tumorigenic spermatogonia [36]. This may explain the TERF1-tsi signal in tumor sample #3. TERF1-tsi was also absent in the TCam-2 cell line, the only available seminoma cell line available (Figure 11C). Together, these data support the idea that TERF1-tsi expression is lost during tumorigenesis. However, it is also likely that seminoma may arise from TERF1-tsi negative cells and thus TERF1-tsi positive spermatogonia may be underrepresented in the tumor samples. Moreover, we could detect TERF1/PIN2 proteins in seminoma tumor samples when pan-TERF1 antibody was used (ab1423). On the other hand, TERF1-tsi expression was not detectable in the seminoma tumor sample when the TERF1-tsi specific antibody was used (AB2), in contrast to its expression in normal testis spermatogonia (Figure 12).

### 2.4. TERF1-tsi Is Expressed in Human CD34-Positive Hematopoietic Stem Cells

**Figure 13:**
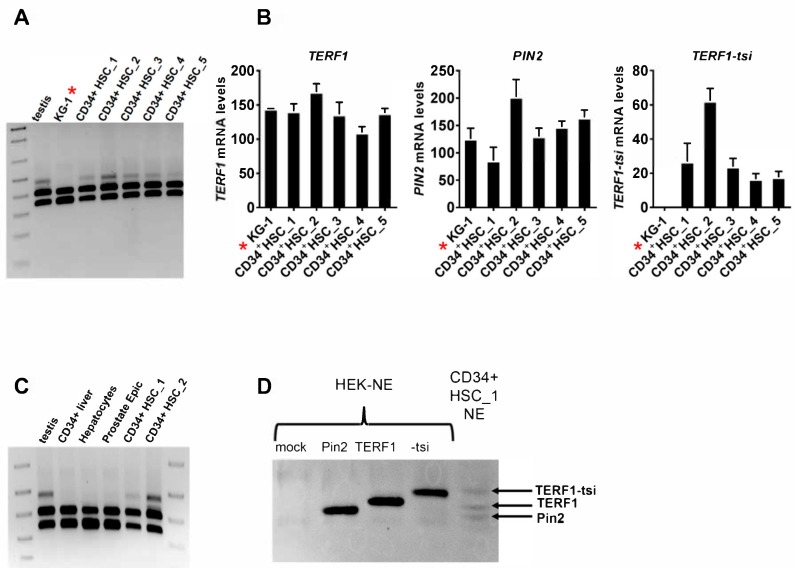
TERF1-tsi expression is detectable in CD34+ human hematopoietic stem cells (CD34+ HSCs). (**A**) Expression of TERF1 isoforms was determined in CD34+ HSCs from five different donors (1 to 5, respectively), along with the testis as a positive control and the KG1 cell line. Semi-quantitative RT-PCR with the pan-*TERF1* primer pair, which amplify all three *TERF1* isoforms, *TERF1*, *PIN2,* and *TERF1-tsi*. * is solely to highlight the KG-1 cell line. (**B**) Real-time quantitative RT-PCR with KG1 cell line and the CD34+ HSCs as in Figure 13A. Isoform-specific primer pairs were used for the qPCR. Real-time RT-qPCR were mean values from two independent experiments with three technical replicates each. Error bars show the mean of SD. (**C**) Semi-quantitative RT-PCR to determine the expression in human CD34+ liver cells, human primary hepatocytes, and human primary prostate epithelial cells with the pan-*TERF1* primer pair, which amplify all three *TERF1* isoforms, *TERF1*, *PIN2*, and *TERF1-tsi*. The testis sample and the indicated human CD34+ HSC samples were included as positive controls. (**D**) Western blot with nuclear extracts prepared from CD34+ HSCs_1 (sample number 1). Nuclear extracts from untransfected and TERF1 isoform transfected HEK cells were used as controls. The indicated protein bands run at the same size as the transfected TERF1 variants. Please note that due to the expected low amounts of TERF1 proteins, CD34+ HSC_1-NE was loaded in excess (60 µg nuclear extract versus 3µg HEK-NE).

In the initial semi-quantitative RT-PCR analysis (Figure 2), we could detect TERF1-tsi only in testis tissue. The above data indicated however that TERF1-tsi expression may be restricted to a subset of stem cells in a given tissue, making it difficult to detect. Thus we analyzed its expression in CD34-enriched population of human hematopoietic stem cells (HSCs), CD34-enriched population of human liver cells, human hepatocytes, human prostate epithelial cells, and the CD34-positive human acute myelogenous leukemia cell line KG-1 [37]. Both semi-quantitative PCR and RT-qPCR data clearly show the presence of *TERF1-tsi* mRNA in the CD34-enriched HSCs (Figure 13). A testis sample was included as a positive control in the semi-quantitative PCR analyses. Interestingly, although *TERF1-tsi* mRNA was detectable in CD34-enriched HSC from all tested five different donors, it was absent in all other cell types as indicated (Figure 13). Again, both semi-quantitative and quantitative RT-PCR analyses indicated that *TERF1* and *PIN2* mRNA levels did not differ substantially among all samples, including the KG1 cells (Figure 13B). This observation is in line with the low/reduced expression of *TERF1-tsi* in the human seminoma samples, as shown in Figure 11B, indicative for a potentially genuine downregulation of *TERF1-tsi* in tumor cells.

To detect endogenous TERF1-tsi in human HSCs at the protein level, nuclear extracts were prepared from one of the human HSC samples where sufficient cells were available (CD34+ HSC_1). As the above experiments with human testis samples clearly showed a nuclear localization of all TERF1 variants, an enrichment of these proteins in the nuclear fraction of human HSCs was expected. In fact, using the pan-TERF1 antibody ab1423, we observed signals at the expected size of the three TERF1 splice variants (Figure 13D). As a control, we used HEK cells that lack endogenous TERF1-tsi. Please note that due to the high transfection efficiency, the ectopic TERF1 proteins were present in excess and thus the endogenous TERF1/PIN2 proteins are not visible in the transfected HEK cells; i.e., the Western blot experiment shows only the ectopic TERF1 variants in the HEK cell line (Figure 13D).

### 2.5. TERF1-tsi Mainly Localizes to Telomeres

Immunohistochemistry experiments indicated that TERF1-tsi is localized to the cell nucleus. To verify this observation in cells and to elucidate whether TERF1-tsi also colocalizes with telomeric DNA or proteins, immunofluorescence (IF) experiments were performed. For this purpose, commercially available TERF1 antibodies (e.g., ab1423) detecting all TERF1 variants and TERF1-tsi specific AB2 antibody were used. Experiments were conducted with ectopic TERF1-tsi and TERF1 in several cell lines (not shown), including U2OS, which were finally chosen for further experiments as these cells possess long telomeres and accumulate higher amounts of telomere-binding proteins enabling their easy detection by IF. As expected, using the ab1423 antibody, endogenous TERF1/PIN2 signals were observed in parental U2OS cells (Supplementary Figure S3A) and TERF1 signals were exclusively localized in the cell nucleus [38,39]. On the other hand, transfected U2OS cells revealed a mixture of endogenous TERF1/PIN2 and ectopic TERF1 or TERF1-tsi signals, which could also be discriminated according to signal intensities (Supplementary Figure S3B–C): transfected U2OS cells showed strong TERF1 signals (ectopic TERF1) compared to untransfected cells (endogenous TERF1/PIN2) (compare to Supplementary Figure S3A–C).

**Figure 14:**
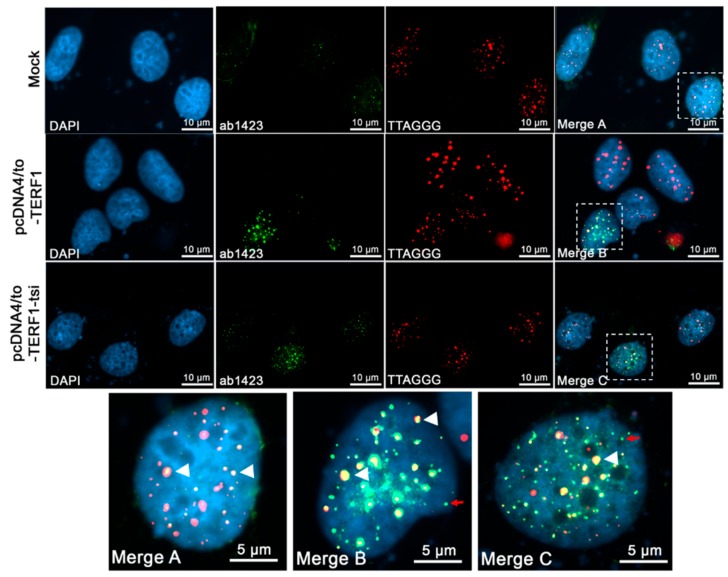
Immunofluorescence telomere-FISH staining to detect endogenous and ectopic TERF1 isoforms/telomere co-localization with a pan-TERF1 antibody, ab1423 (1:300). From left to right, (first column) nuclei were counterstained with the blue fluorescent DNA dye, DAPI. (Second column) ab1423 staining in mock and transfected cells showing signals correspond to either endogenous TERF1/PIN2 in parental (mock-transfected) U2OS cells or ectopic TERF1 and TERF1-tsi (untagged TERF1 or TERF1-tsi in pCDNA-4TO vector) in transfected U2OS cells, respectively. (Third column). Cy3-conjugated telomere-specific peptide-nucleic-acid (PNA) probe, red fluorescence revealing telomeres signals in parental and transfected U2OS cells. (Fourth column and bottom row) Merged images of DAPI, ab1423 staining, and telomere PNA-FISH signals. The selected nuclei (fourth coloumn) were enlarged (bottom row) for better visualization. The endogenous TERF1/PIN2 and ectopic TERF1 and TERF1-tsi revealed co-localization with the telomeric probe in parental and transfected U2OS cells (white arrowheads). Interestingly, subset of ectopic TERF1 and TERF1-tsi signals showed no co-localization with telomeres (red arrows). All images were captured with ApoTome at 630×. Scale bars (5 µm or 10 µm) are indicated.

**Figure 15:**
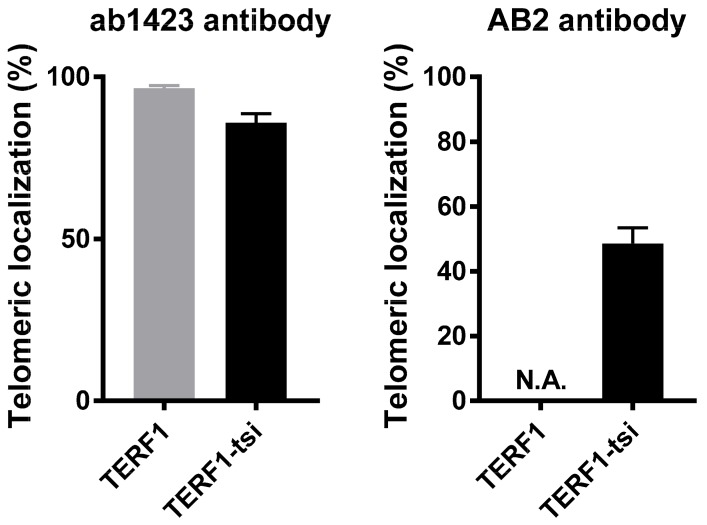
Quantification of telomeric localization of TERF1 and TERF1-tsi. (Left) Quantification of telomeric TERF1 or TERF1-tsi signals detected by the panTERF1 antibody ab1423 and the telomeric FISH. Colocalization of TERF1 or TERF1-tsi with telomeric DNA was evaluated in at least 20 nuclei in each independent repeat experiment. The values indicate percent co-localization of TERF1 or TERF1-tsi with the telomeric DNA signal. (Right) Quantification of telomeric TERF1-tsi signals detected by the TERF1-tsi specific antibody AB2 and the telomeric FISH. Colocalization of TERF1-tsi with telomeric DNA was evaluated in at least 20 nuclei each in 4 independent repeat experiments. The values indicate percent co-localization of TERF1-tsi with the telomeric DNA. N.A. (not applicable): AB2 did not show unspecific cross-reaction with TERF1.

The ectopic expression of TERF1 variants was also analyzed for telomeric co-localization by combined IF/qFISH (quantitative fluorescence in situ hybridization for telomeric DNA using fluorescently labeled peptide nucleic acid probe). In line with previous reports, endogenous TERF1/PIN2 localized to telomeres (Figure 14, top row: mock-transfected cells). Also, TERF1-overexpressing U2OS cells revealed strong telomeric localisation (~96% co-localized signals) of the endogenous and ectopic TERF1 (Figure 14, middle row; Figure 15, left). Importantly, analysis of TERF1-tsi with the ab1423 antibody also showed significant co-localization with telomeres (~86% co-localized signals) (Figure 14, third row; Figure 15, left). Of note, while the endogenous TERF1/PIN2 showed nearly complete telomeric co-localization, the ectopic TERF1 and TERF1-tsi revealed unanticipated non-telomeric dots (Figure 14, bottom row, dots indicated by red arrows in enlarged nuclei; Figure 15, left), likely due to the strong overexpression of these proteins (see discussion in Section 3).

**Figure 16:**
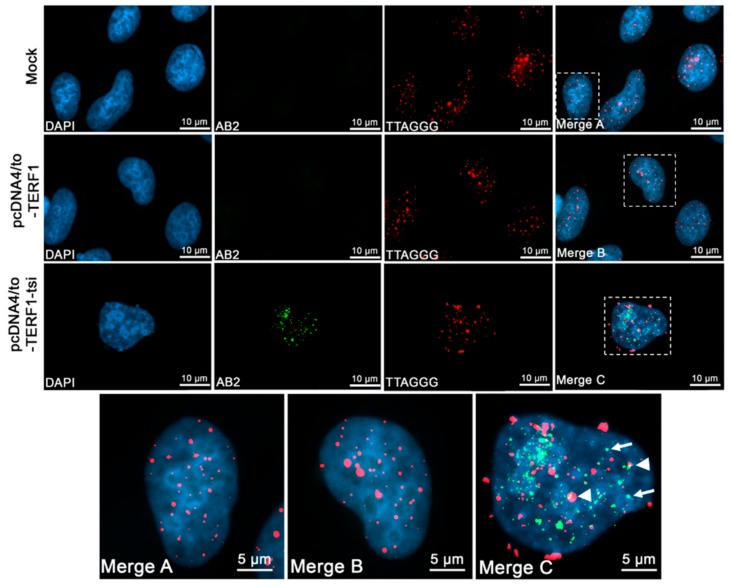
Immunofluorescence telomere-FISH staining to detect the telomeric localisation of ectopic TERF1-tsi in pcDNA4/to-TERF1-tsi transfected U2OS cells with TERF1-tsi specific antibody AB2 (1:100). Secondary antibody Alexa488 (1:2000). From left to right, (first column) nuclei were counterstained with the blue fluorescent DNA dye, DAPI. (Second column) AB2 staining in mock and transfected cells showing ectopic TERF1-tsi specific signals (bottom) in transfected U2OS cells. (Third column). Cy3-conjugated telomere-specific peptide-nucleic-acid (PNA) probe, red fluorescence revealing telomeres signals in parental and transfected U2OS cells. (Fourth column and bottom row) Merged images (DAPI, AB2 staining, and telomere PNA-FISH) of single nuclei of mock (merge A), TERF1 (merge B), and TERF1-tsi (merge C)-transfected cells. AB2 antibody detects TERF1-tsi (second column, lower lane) but not endogenous or ectopic TERF1/PIN2 (second column, top or middle lane) confirming that AB2 has no cross-reactivity with endogenous TERF1/PIN2 and ectopic TERF1. Importantly, AB2 staining of TERF1-tsi revealed partial co-localization with the telomeric probe (merge C; white arrowheads) while a subset of TERF1-tsi did not show telomeric colocalization (white arrows). All images were captured with ApoTome at 630x. Selected nuclei were enlarged for better visualization of the signals. Scale bars (5 µm or 10 µm) are indicated.

Telomeric and nuclear localization of TERF1-tsi was also evaluated by the isoform-specific antibody, AB2, in combination with telomere-FISH. Importantly, IF-signals were only detectable in TERF1-tsi transfected cells (Figure 16, bottom row; enlarged nuclei) but not in mock-transfected or TERF1-transfected cells (Figure 16, top and middle row), indicating the specificity of the AB2 for IF. However, the analyses also revealed only a partial TERF1-tsi/telomere co-localization signals when AB2 was used to detect TERF1-tsi and telomere signals (Figure 15 and Figure 16, right). This result indicates that the AB2 does not detect all telomeric TERF1-tsi molecules, maybe due to hindrance by protein–protein interactions which may mask the access of the antibody to the target epitope.

**Figure 17:**
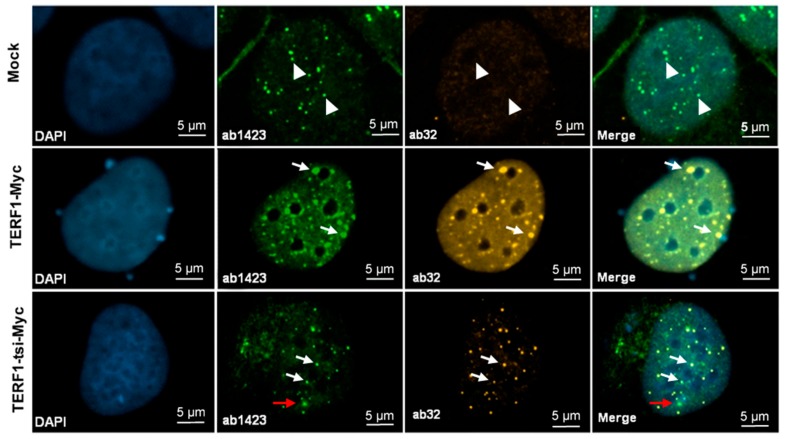
Detection of endogenous TERF1/PIN2 and ectopic Myc-tagged-TERF1 and-TERF1-tsi by immunofluorescence. From left to right, (first column) nuclei were counterstained with the blue fluorescent DNA dye, DAPI. (Second column) ab1423 staining of mock and transfected cells showing positive signals in all cells. (Third column) ab32 staining of mock and transfected cells showing positive Myc-tag signals in transfected cells. (Fourth column) Merged images of DAPI, ab1423, and ab32. The top row shows only positive, endogenous nuclear TERF1/PIN2 with ab1423 (white arrowheads) but no cross-reactivity with the anti-Myc antibody. In the middle row, endogenous TERF1/PIN2 and Myc-TERF1 signals show complete co-localization with ab1423/ab32 antibodies (white arrowheads). Similarly, bottom rows show overlapping ab1423/ab32 signals which correspond to the ectopic Myc-tagged TERF1-tsi in the indicated Myc-TERF1-tsi transfected cells. These overlapped signals discriminate the ectopic Myc-tagged TERF1-tsi from endogenous TERF1/PIN2. Here, few TERF1/PIN2 signals without TERF1-tsi can be observed (red arrow). The images were captured at 630x. The bar indicates 5µm. Secondary antibodies Alexa488 (1:2000) for ab1423, and Cy3 (1:1000) for ab32.

**Figure 18:**
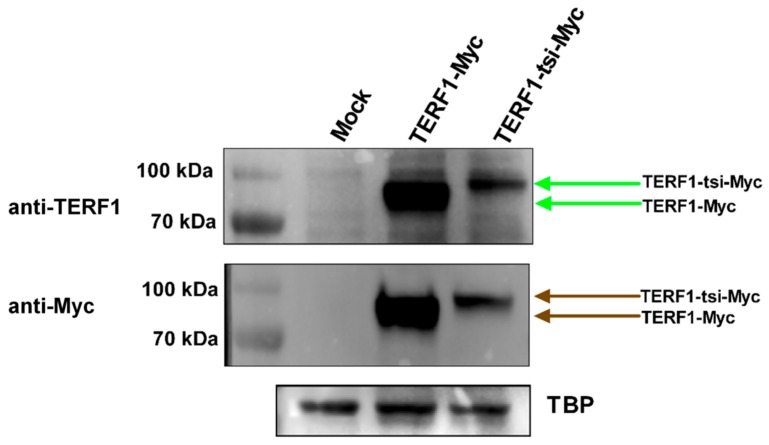
Detection of ectopic Myc-tagged-TERF1 and -TERF1-tsi in nuclear lysates of transfected U2OS cells. (Top) Immunoblot with a pan-TERF1 antibody ab1423 (1:300). Due to the Myc epitope fusion (6x-Myc), the Myc-TERF1 and Myc-TERF1-tsi signals appear at ~90 kDa. Due to strong ectopic expression of the respective TERF1 variants, the endogenous TERF1/PIN2 are not clearly visible (Middle) Immunoblot with Myc-tag antibody ab32 (1:500) showing specific signals at ~90 kDa. (Bottom) Loading control using an antibody against TATA-binding protein (TBP). Of note, despite equal loading, immunoblot results with both antibodies repeatedly confirmed that the ectopic TERF1-tsi was less abundant than TERF1, indicating a post-translational regulation of the protein stability.

In the above described experiments, we observed that a subset of ectopic TERF1 and TERF1-tsi signals showed no co-localization with telomeres, whereas endogenous TERF1/PIN2 were detected only at telomeres. In order to clarify whether (i) the non-telomeric TERF1 signals were due to the high protein levels following the ectopic expression, and (ii) TERF1 and TERF1-tsi co-localize, U2OS cells were transfected with vectors encoding Myc-TERF1 or Myc-TERF1-tsi and analyzed with an antibody (ab32) that detects the Myc-epitope specifically in combination with the TERF1 antibody ab1423 (Figure 17). Importantly, both TERF1 and TERF1-tsi transfected cells show telomeric dot-like structures with the anti-Myc antibody (Figure 17, middle and bottom row) whereas no signals were detected in mock-transfected cells (Figure 17, top row). Although anti-panTERF1 antibody detects both untagged and tagged TERF1 isoforms, some ectopic Myc-TERF1-tsi signals can be detected which do not co-localize with the signals obtained with the anti-panTERF1 antibody (Figure 17, red arrow). This may indicate that forced ectopic expression of the TERF1 isoforms can result in mislocalization of these proteins. The expression of Myc-tagged proteins was verified by Western blot analysis using nuclear extracts of the transfected U2OS cells (Figure 18).

**Figure 19:**
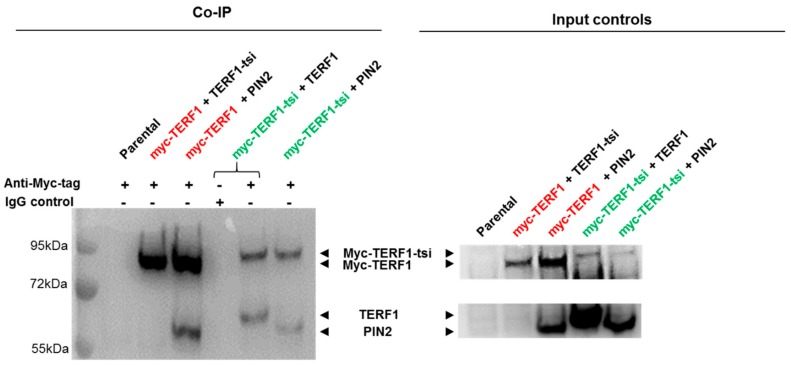
Potential interaction of endogenous or ectopically co-expressed Myc-tagged or untagged TERF1 isoforms was determined co-immunoprecipitation (Co-IP) experiments. The use of anti-Myc- tag antibody or the IgG control for the IP is indicated by “+” or “-“, respectively. Nuclear lysates from parental and transfected HEK cells were used for the Co-IP (left) and input samples (right). The anti-Myc antibody ab32 or the the IgG control were used for the IP. The indicated proteins were detected by Western blot using panTERF1 antibody ab10579. Please note that two areas of the same membrane are divided into upper and lower parts of the figures for better visualization (top: myc-tagged isoforms, ~90 kDa; and bottom: untagged isoforms, ~60 kDa). The combinations of transfected vectors (either Myc-tagged or untagged) are indicated above the blots. Myc-tagged constructs are indicated by red an green font and untagged constructs are indicated by black font. Exposure time for immunoblot: 2 seconds.

To provide further evidence for the potential interactions between TERF1-tsi and the ubiquitously expressed TERF1 isoforms, we used co-immunoprecipitation as an independent experimental approach (Figure 19). We show that Myc-TERF1-tsi can pull-down co-transfected untagged TERF1 or untagged PIN2. The results of these experiments confirm the interaction of TERF1-tsi with TERF1 or PIN2.

In conclusion, the results provide evidence that TERF1-tsi mainly localizes to telomeres (co-localization with endogenous TERF1/PIN2), and likely forms heterodimers with TERF1/PIN2. Another interesting observation was, based on the Western blot analyses (see Figure 10, Figure 18 and Figure 19), that ectopic TERF1 was consistently more abundant than the ectopic TERF1-tsi, potentially indicating a post-translational regulation of TERF1-tsi to maintain low TERF1-tsi at the protein level.

## 3. Discussion

TERF1 is an ubiquitously expressed protein and has crucial functions in telomere maintenance and cell cycle regulation while essential for organismal viability [40,41,42]. A TERF1 splice variant, PIN2, was independently discovered as a protein that can bind the mitotic kinase NIMA and suppress its lethal phenotype [28]. Later, it was shown that PIN2 is a TERF1 splice variant, lacking the exon 7 of *TERF1* gene (corresponding to a 20 amino acid sequence) [29]. Here we describe the identification of a novel stem-cell and tissue-specific TERF1 isoform, TERF1-tsi. A new exon (exon 9) with conserved intron-exon recognition sequences progressively evolved between exon 8 and exon 10 to give rise to a 90 base-pair long additional splice variant of *TERF1* gene. Genome sequence comparison revealed the evolution of new intron-exon boundaries (AG-GT) and of the new exon, which are conserved among hominidae. Here, we could experimentally show the expression of a new splice variant in human and chimpanzee. Interestingly, Hartmann and Scherthan mentioned an additional TERF1 splice variant in human testis although no sequence structure and product size were shown [43]. It is likely that the authors observed the TERF1-tsi splice variant but did not further characterized it. Of note, using transgenic animal models, it was shown that TERF1 is a stem cell factor [44]. While *Terf1/Pin2* can be detected in mouse tissues, so far, all analyses related to Trf1 function in mouse are attributed to the main splice variant Trf1. It is tempting to speculate that the different functions inherent to mouse Trf1 are executed by different TERF1 splice variants in hominidae.

By immunohistochemistry with two newly established, isoform-specific (TERF1-tsi) antibodies directed against the peptide fragments of exon 9, we show that expression of TERF1-tsi is restricted to a subset of spermatogonia in the human testis. Previous studies identified three nuclear subtypes of spermatogonia, A-dark, A-pale, and B-type, with distinct markers that define these spermatogonia (e.g., GPR125, SSX2-4) [45,46,47,48]. Unfortunately, our TERF1-tsi antibodies were unsuitable for co-immunostaining with these markers in initial experiments (not shown). Currently, we cannot assign TERF1-tsi expression to a certain subtype of spermatogonia and we do not have a comprehensive explanation of why only a subset of the spermatogonia stain positive for TERF1-tsi. These questions remain to be elucidated in future studies, potentially by co-immunostaining of TERF1-tsi with other markers, or by gene expression and functional analyses in spermatogonial subtypes that were enriched by specific surface markers. Another important area of future studies will include more in-depth studies to address the potential expression of TERF1-tsi in other tissue stem cells, e.g., intestinal or skin stem cells.

Our study also revealed that *TERF1-tsi* expression was low or absent in human seminomas, testicular germ cell tumors likely arising from spermatogonia [49]. Moreover, *TERF1-tsi* expression was also absent from the established seminoma cell line, TCam-2. These observations allow at least two interpretations. Either testicular tumors arise from TERF1-tsi negative spermatogonia, or alternatively, TERF1-tsi expression is downregulated during tumorigenesis. It remains to be elucidated in future studies which of these two possibilities holds true or whether there is another explanation for these observations. For this, on one side, a large number of testicular tumor samples of different origins needs to be analysed. On the other hand, in the absence of cultured cells with endogenous TERF1-tsi, ectopic expression of this splice variant in TCam-2 cells will be a suitable tool to gain further insights into TERF1-tsi in cell proliferation and tumor suppression. Interestingly, we also observed the expression of TERF1-tsi in (healthy)-donor-derived CD34+-HSCs but not in the CD34+-KG1 cell line (Figure 13) or other leukemia cell lines such as HL60 or U937 (unpublished observations). It would be interesting to find out whether TERF1-tsi is expressed in a subset of human CD34+ HSC population and whether its expression is downregulated during tumor progression. Besides, KG1 cells, in addition to the TCam-2 cells offer another option for functional studies to address the role of TERF1-tsi in cancer. A downregulation of TERF1 in gastric cancers was reported [50]. In contrast, an upregulation of TERF1 expression was reported in some human epithelial cancers [35,51,52]. Although we did not do an in-depth analysis in this respect, we consistently observe higher PIN2 mRNA levels in human established cell lines compared to the TERF1 splice variant (unpublished observation). Irrespective of the role of TERF1-tsi in stem-cell-derived cancers, it is therefore important to determine the differential expression of the TERF1 splice variants in the cancer context. Also, our semi-quantitative expression analyses in human tissue samples indicate potential tissue-specific and functional differences of the TERF1 splice variants.

Our analyses indicate that TERF1-tsi is a nuclear protein, mainly localized at telomeres. Although we also observe few non-telomeric TERF1-tsi signals when the pan-TERF1 antibody ab1423 was used for immunofluorescence, it is likely that this is due to strong ectopic expression of the protein, as we also observe similar non-telomeric TERF1 with this antibody. This conclusion is supported by the IF and co-IP results obtained with the myc-tagged TERF1 isoforms. The non-telomeric TERF1-tsi was, however, more prominent when the TERF1-tsi specific antibody AB2 was used. One potential explanation for this observation is that the epitope may be masked by protein–DNA or protein–protein interactions at the telomeres. This would, however, mean that non-telomeric TERF1 exists in the cell which is not detected by the commercial antibodies. Although we cannot exclude unspecific reactivity of the AB2 antibody, we do not observe any cross-reactivity in TERF1 transfected cells or empty vector-transfected control cells. This obscure observation will be addressed in future studies. We also wish to point out here that the presented studies were performed in the telomerase-negative ALT (alternative lengthening of telomeres) cell line U2OS, due to high telomere signal intensities in this cell line. Potential telomeric/non-telomeric functions of TERF1-tsi in telomerase positive cells remain to be elucidated in further studies. In this context, TERF1 was shown to negatively regulate telomere length by inhibiting telomerase-dependent elongation [53] and interestingly, it was speculated that the telomere replication is facilitated by TERF1 to prevent replication fork-stalling, which causes telomeric replication stress [27,54]. It would be interesting to elucidate the potential role of TERF1-tsi in telomere-replication or preventing telomere fragility in stem cells.

## 4. Materials and Methods

### 4.1. Plasmids

Plasmids vectors pcDNA4/to-TERF1, pcDNA4/to-TERF1-tsi, pcDNA4/to-PIN2 were generated by EcoRI-cloning of the PCR amplification products from human testis cDNA into pcDNA4/to empty vector. For the pCS2 + 6 × Myc-TERF1, and pCS2 + 6 × Myc-TERF1-tsi vectors, the respective inserts were subcloned from pCDNA4/to by EcoRI digestion into the PCS2+ vector in frame with the 6xMyc-epitope.

### 4.2. Cell Lines

HEK293 (adherent epithelial cells of human embryonic kidney/ ATCC® CRL-1573™) and U2OS (adherent osteosarcoma cells from human bone/ ATCC® HTB-96™) cell lines were provided by Research Laboratory of the Department for Urology at Ulm University Hospital; they were cultured in DMEM supplemented with 10% fetal calf serum (FCS) (Biochrom, Karlsruhe, Germany) and 1% penicillin/streptomycin (Invitrogen, California, USA). TCam-2 cells (provided by Prof. Hubert Schorle, Bonn, Germany) were maintained in RPMI supplemented with 10% FCS and 1% penicillin/streptomycin. Cells were grown in incubators maintained at 37 °C and 5% CO2. The cells were fed and split twice per week. HEK293 and U2OS cells were transfected with the aid of transfection reagent PEI (polyethyleneimine) Polysciences Europe GmbH, Hirschberg, Germany) according to the standard procedures.

### 4.3. Human and Mouse Samples

Human normal and tumor testes tissues were provided by the Department for Urology at Ulm University Hospital, and the samples were obtained from patients and the procedures complied to the ethical standards approved by the Ethics Committee of Ulm University with ethical approval number 368/16. Chimpanzee (*P. troglodytes*) testis sample was provided by the Biomedical Research Centre (Rijswijk, Netherlands) and the testes RNA from *M. mulatta* and *C. jacchus*) were kindly provided by the German primate center (Deutsches Primatenzentrum, Göttingen, Germany). Pathological human testis samples were kindly provided by Heike Cappallo-Obermann and Christiane Kirchhoff (University Hospital Hamburg-Eppendorf, Germany). Ethics committee approval (OB/X/2000) was obtained and the studies were conducted in accordance with the guidelines of the Helsinki Declaration. Human CD34+ cells stimulated with G-CSF were prepared by leukapheresis. Informed consent was taken from all patients and approved by the local ethics committee. Immunomagnetic selection of CD34+ cells was performed using the CliniMACS® system (Miltenyi Biotech GmbH, Germany). Flow cytometry (FACS) analysis using anti-CD34 antibody conjugated with phycoerythrin (PE) (Becton Dickinson GmbH, Germany) showed a purity of more than 98% for CD34+ HPC. Mouse testes tissues were prepared from C57BL/6 mice and the procedures complied to the ethical standards approved by the Ethics Committee of Ulm University with ethical approval number 1326.

### 4.4. Immunohistochemistry (IHC)

Human and mouse testis tissues were embedded in paraffin, and 3 µm sections were prepared for immunohistochemical analyses, and IHC was performed as described previously [55]. Following antibodies were used for IHC: anti-TERF1 antibody (ab1423), at 1:100 dilution, and anti-TERF1-tsi (AB2), at 1:100, 1:200, and 1:400 dilutions, respectively.

### 4.5. Western Blot

Western blot and the preparation of cell lysates were performed as described [56]. In brief, same numbers of mock-transfected and TERF1-isoform transfected cells were used to extract cytoplasmic and nuclear proteins in Buffer A (10 mM Hepes pH 7.9, 1.5 mM MgCl2, 10 mM KCL, 1 mM DTT, 0.5 mM PMSF) and Buffer B (20mM HEPES pH 7.9, 420 mM KCl, 1.5 mM MgCl2, 1 mM DTT, 25% glycerol, 0.5 mM PMSF), respectively. Equal protein amounts were loaded and proteins were separated by SDS-PAGE and transferred to PVDF membranes for immunoblotting.

### 4.6. Co-Immunoprecipitation

Three hundred µg of nuclear extracts from mock- or TERF1-transfected cells were used. For each lysate, 3 µg of anti-Myc antibody or rabbit IgG were incubated along with protease inhibitors. The reaction was placed on rotation at 4 °C for 2 h. Then, the mixture was added to the Pierce™ protein A agarose beads and rotation was continued for another hour. Afterward, the beads were pelleted by centrifugation and carefully washed three times with 500 µL buffer (20mM Tris pH 8.0, 100 mM KCl, 0.2 mM EDTA, 5 mM MgCl2, 10% glycerol, and 1% NP-40. Subsequently, the beads were resuspended with 27 µL nuclear extraction buffer (20 mM HEPES, pH 7.9, 25% glycerol, 0.4 M NaCl, 1 mM EDTA, 0.5 M DTT, and protease inhibitor cocktail).

### 4.7. Immunofluorescence and Telomere FISH

U2OS cells were seeded on coverslips in a 6-well plate; cells were transfected with 1 µg of the respective vectors and stainings were performed 48 h post-transfection at 70%–80% cell density.

Immunofluorescence in combination with Telomere-FISH was adapted from [57] and performed with few modifications as described [58].

### 4.8. RNA Isolation and RT-PCR

Total RNA was either prepared from fresh frozen tissue samples or cultured human cells. RNA isolation and RT-PCR and real-time RT-PCR were performed as described previously [59]. The human Total RNA Master Panel II (Cat. #636643) was purchased from Clontech (now Takara Bio Inc.), the CD34 positive (CD34+) liver cells were purchased from PELOBiotech GmbH (Martinsried, Germany), the human prostate epithelial cell total RNA (Catalog #4415) and the human hepatocyte total RNA (Catalog #5205) were obtained from ScienceCell Research Laboratories (Carlsbad, CA, USA). Human normal testis/testicular cancer total RNA samples (#CR562185; #CR562186, #CR561785, #CR562933 #CR561980, #CR561981) were purchased from OriGene Technologies, Inc. (Rockville, MD, USA).

### 4.9. Primer Sequences

The following primer combinations were used to amplify TERF1 splice variants.

For the amplification of TERF1/PIN2/TEFR1-tsi in one reaction: 

hmsFor: 5’-tcaacctttctaatgaaggcagc-3’ and hmsRev: 5’-tgtcttcttcccaaagccatg -3’

These primer sequences are conserved in human, mice and sheep, hence the abbreviation: hms.

For TERF1 splice-variant-specific semi-quantitative and quantitative RT-PCR:

TERF1-tsi:

hTERF1-tsi-F1: 5’-atgaataagaaagaaggagtgagg-3’ and hms Rev: 5’-tgtcttcttcccaaagccatg-3’

TERF1:

TERF1-iso-F1: 5’-tgttagtgacaaacagtctgcgg-3’ and TERF1-iso-R: 5’-cttttttcttttttgtactttgaggag-3’ 

PIN2:

PIN2-iso-F1: 5’-ttggatacaagaaaaaggtctcac-3’ and hms Rev: 5’-tgtcttcttcccaaagccatg-3’

Primer combinations for the housekeeping genes.

GAPDH: GAPDH-F: 5’aaggtcatccctgagctgaac-3‘ and GAPDH-R: 5‘-acgcctgcttcaccaccttct

S27 Ribosomal protein: S27-F: 5’-aacatgcctctcgcaaagga-3’ and S27-R: 5’-tgtgcatggctaaagaccgt-3’.

### 4.10. Antibodies

Anti-TERF1 antibody—ChIP Grade (ab1423) (Abcam, Cambridge, United Kingdom)—was used at 1:100 dilution for IHC, 1:300 for IF and 1:500 for WB. Anti-Myc-tag antibody [9E10]—ChIP Grade (ab32) (Abcam, Cambridge, UK)—was used at 1:500 dilution for WB and 1:500 for WB. Anti-TERF1-tsi (AB2) polyclonal antibody (Davids Biotechnologie GmbH, Regensburg, Germany) was used at 1:100, 1:200, and 1:400 for IHC, 1:100 for IF and 1:100, and 1:200 for WB. Anti-β-Actin monoclonal antibody produced in mouse (Sigma Aldrich, Missouri, USA) was used at 1:10,000 for WB. Anti-TBP antibody produced in goat (Sigma Aldrich, Missouri, USA) was used at 1:1000 in WB. Secondary antibodies: goat anti-rabbit IgG (H+L) highly cross-adsorbed secondary antibody, Alexa Fluor 488 (Invitrogen, California, USA) 1:2000 for IF. Cy™3 AffiniPure F(ab’)_2_ fragment goat anti-mouse IgG (H+L) (Jackson ImmunoResearch, Cambridgeshire, UK) was used at 1:1000 for IF.

### 4.11. Generation of TERF1-tsi Specific Antibodies

To produce antibodies against TERF1-tsi, two rabbits were immunized with two synthetic peptides, CLTKLTMNKKEGVRK and KKEGVRKGLCFLVLLKHY, according to the 63 Day Antisera protocol by Davids Biotechnologie GmbH, Germany. One of these antibodies, AB2 against the peptide KKEGVRKGLCFLVLLKHY, proved to be best and was used in this study. Independently, AB1 was by ProSci Inc. (Poway. CA, USA) produced against the 30 amino acid peptide (ITYICLTKLTMNKKEGVRKGLCFLVLLKHY) representing exon 9. The third bleed from two immunized rabbits was then combined, affinity purified, and positively tested by ELISA.

### 4.12. Software

Zen2 imaging software, version 2.3 was used for microscopy imaging in the IHC and IF experiments (Carl Zeiss Microscopy GmbH, Jena, Germany). ImageJ2 was used for relative quantification of Western blots.

## Supplementary Materials

Supplementary materials can be found at https://www.mdpi.com/1422-0067/21/1/85/s1.
