# Supplementary material for: A Novel Tissue and Stem Cell Specific TERF1 Splice Variant Is Downregulated in Tumour Cells
Source: Int J Mol Sci. 2019 Dec 20;21(1):85. doi: 10.3390/ijms21010085 (PMC6981981; doi:10.3390/ijms21010085)
Supplement: Supplementary file 1 [file ijms-21-00085-s001.pdf]

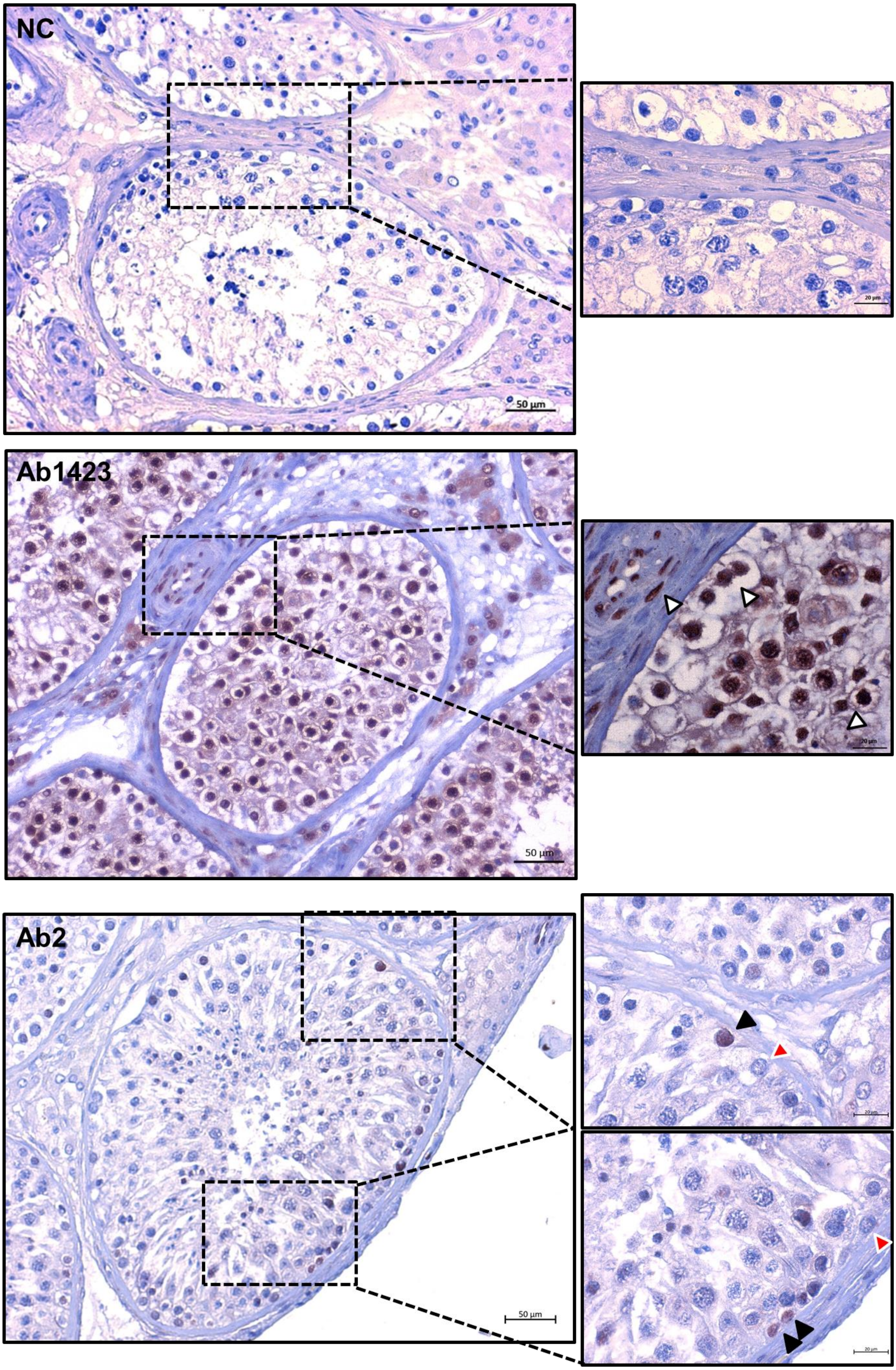
NC
50 µm
Ab1423
50 µm
Ab2
50 µm

Supplementary Figure 1. Immunohistochemical analyses of TERF1 isoforms in normal human testis. (TOP) Negative control. Only secondary antibody. The left image was captured at 200x, and the right one at 630x. (Middle) Immunohistochemical detection of TERF1/PIN2 in human testis section with a pan-TERF1 antibody, ab1423. The figure is a representative IHC with Ab1423 of a human testis section, showing single seminiferous tubule, Leydig (interstitial) cells, and surrounding muscle cells. The staining pattern showed and confirmed the ubiquitous expression of TERF1/PIN2 in all testis cells, including seminiferous tubule's cells, Leydig cells, and surrounding muscle cells. Selected area was magnified to visualize the stains more clearly. The observed staining pattern confirmed that all cells of seminiferous tubules express TERF1/PIN2 (white arrowhaeds with black border). The section was counterstained with hematoxylin. The left image was captured at 200x, and the right one at 630x. (Bottom) Immunohistochemical detection of TERF1-tsi in human testis section with a specific TERF1-tsi antibody, AB2 (1:400). This figure demonstrates a representative testis section through a single seminiferous tubule, Leydig (interstitial) cells, and surrounding muscle cells. AB2 showed strong positive staining, specifically localised in spermatogonia as shown in the left image. For better visualization of the stains, two selected areas were examined at higher magnification as shown in the right images depicting the positively stained spermatogonia (black arrowheads). Of note, some of the spermatogonia did not show any staining (red arrowheads with white border). This section was counterstained with hematoxylin. The left image was captured at 200x, and the right ones at 630x.

TERF1-tsi is not expressed in mouse testis:

NC **Ab2**

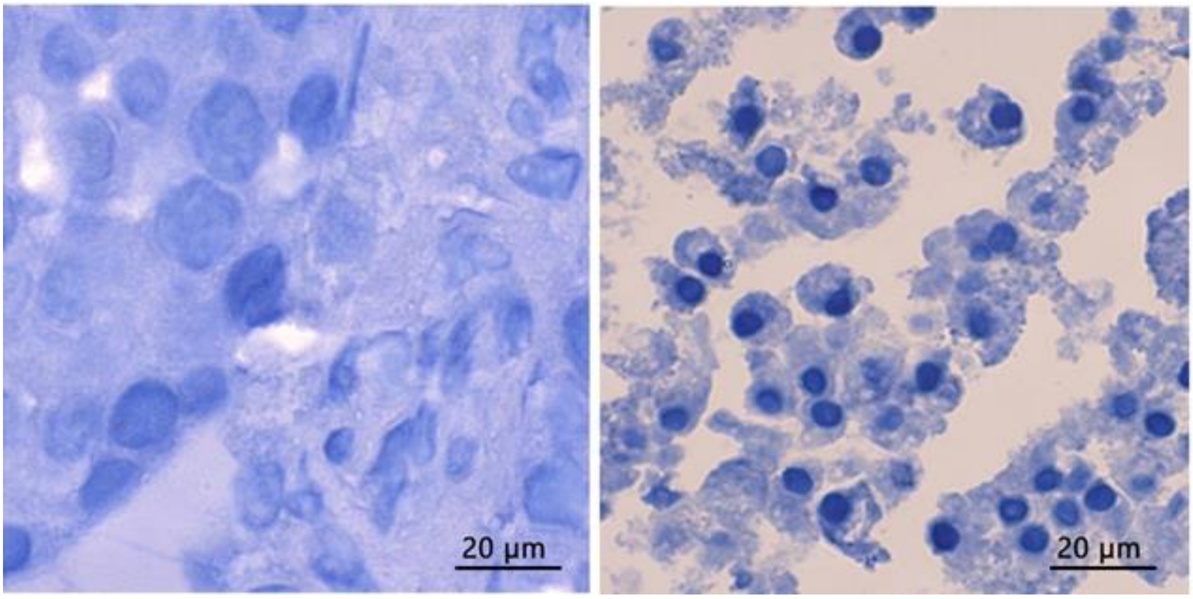

Supplementary Figure 2. Immunohistochemical analyses of TERF1 isoforms in normal mouse testis.

Suppl. Figure 3: TERF1-tsi mainly localizes to telomeres

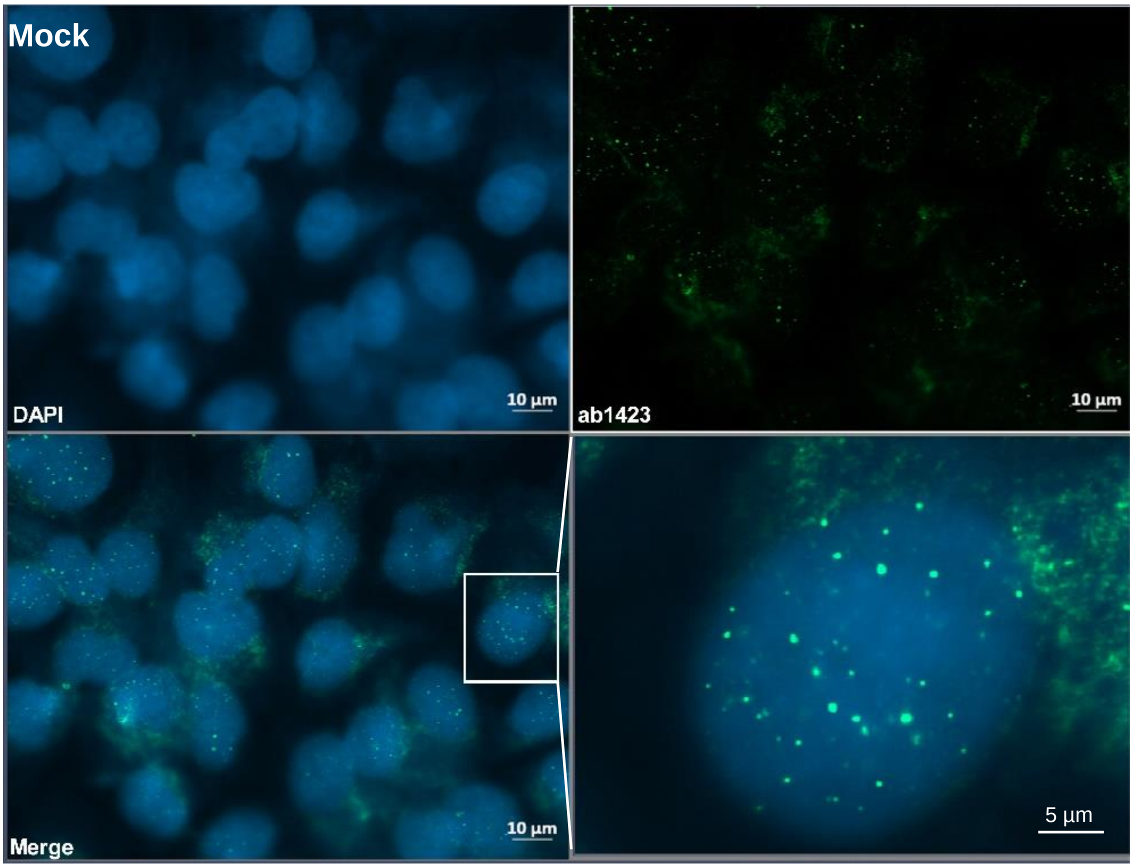

Supplementary Figure 3A. Immunofluorescence detection of endogenous TERF1/PIN2 in parental (mock-transfected) U2OS cells with pan-TERF1 antibody, ab1423 (1:300). (Top-left) Nuclei were counterstained with the blue fluorescent DNA dye, DAPI. (Top-right) ab1423 staining of mock-transfected cells showing positive endogenous TERF1/PIN2 signals. (Bottom-left) Merged image of DAPI and ab1423 staining revealing the nuclear localisation of endogenous TERF1/PIN2 signals. (Bottom-right) The selected nucleus was enlarged for better visualisation, showing the positive signals of endogenous TERF1/PIN2 and their localisation in cell nucleus. The Images were captured at 630x. Secondary antibody Alexa488 (1:2000).

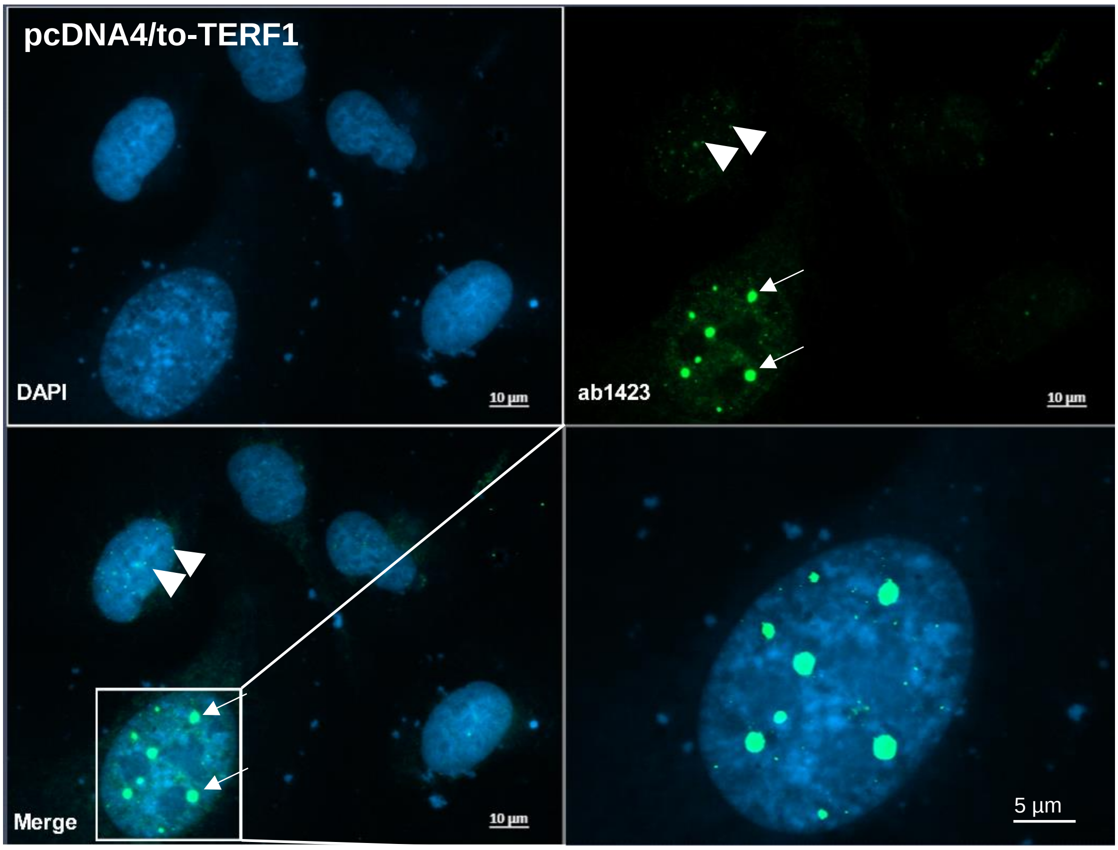

Supplementary Figure 3B. Immunofluorescence detection of endogenous TERF1/PIN2 and ectopic TERF1 in pcDNA4/to-TERF1 transfected U2OS cells with pan-TERF1 antibody, ab1423 (1:300). (Top-left) Nuclei were counterstained with the blue fluorescent DNA dye, DAPI. (Top-right) ab1423 staining of TERF1-transfected cells showing a mixture of positive endogenous TERF1/PIN2 signals (white arrowheads), and ectopic TERF1 signals (white arrows). (Bottom-left) Merged image of DAPI and ab1423 staining revealing the nuclear localisation of the endogenous TERF1/PIN2 and the ectopic TERF1. (Bottom-right) Selected transfected cell nucleus was enlarged for better visualisation revealing the relatively high signal intensity of the ectopic TERF1 compared to the endogenous TERF1/PIN2. The Images were captured at 630x. Secondary antibody Alexa488 (1:2000).

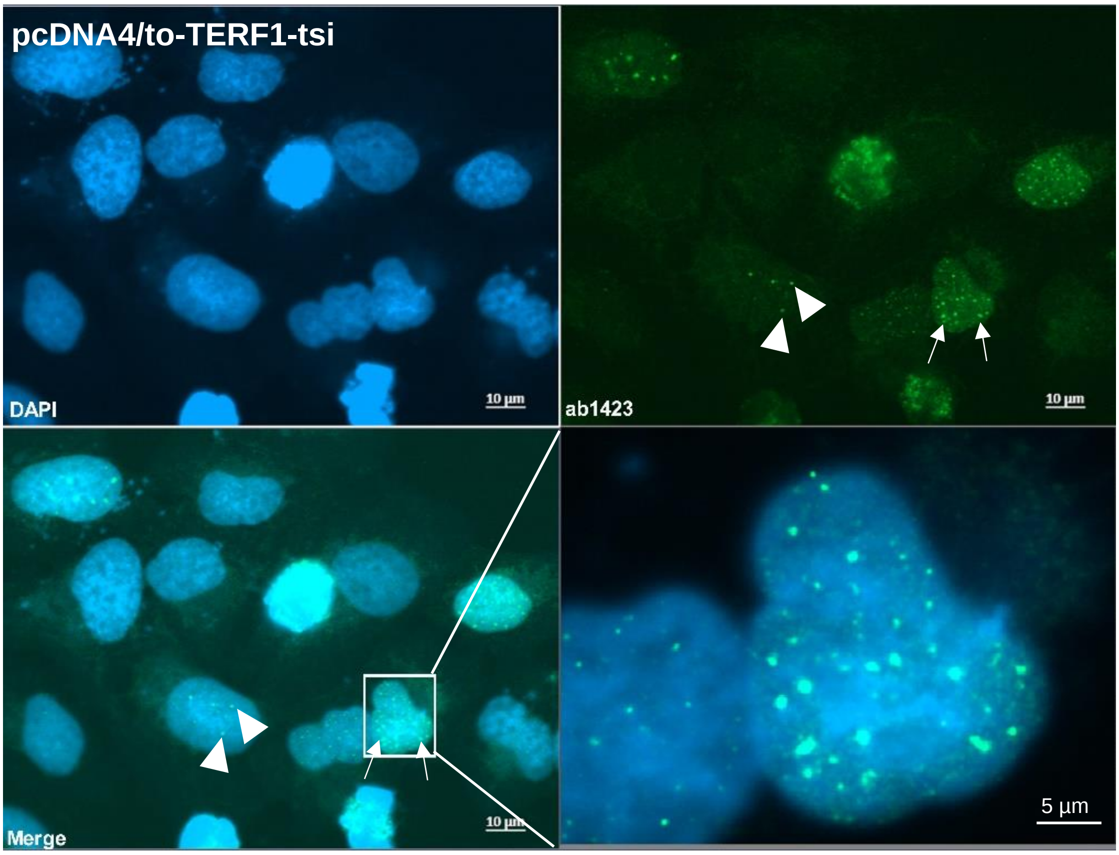

Supplementary Figure 3C. Immunofluorescence detection of endogenous TERF1/PIN2 and ectopic TERF1-tsi in pcDNA4/to-TERF1-tsi transfected U2OS cells with pan-TERF1 antibody, ab1423. (Top-left) Nuclei were counterstained with the blue fluorescent DNA dye, DAPI. (Top-right) ab1423 staining of TERF1-tsi transfected cells showing a mixture of positive endogenous TERF1/PIN2 signals (white arrowheads), and ectopic TERF1-tsi signals (white arrows). (Bottom-left) Merged image of DAPI and ab1423 staining revealing the nuclear localisation of the endogenous TERF1/PIN2 and the ectopic TERF1-tsi. (Bottom-right) Selected transfected cell nucleus was enlarged for better visualisation revealing the relatively high signal intensity of the ectopic TERF1-tsi compared to the endogenous TERF1/PIN2. The Images were captured at 630x. Secondary antibody Alexa488 (1:2000).
